# Sequential Exposure to Obesogenic Factors in Females Rats: From Physiological Changes to Lipid Metabolism in Liver and Mesenteric Adipose Tissue

**DOI:** 10.1038/srep46194

**Published:** 2017-04-07

**Authors:** Marta G. Novelle, María J. Vázquez, Juan R. Peinado, Kátia D. Martinello, Miguel López, Simon M. Luckman, Manuel Tena-Sempere, María M. Malagón, Rubén Nogueiras, Carlos Diéguez

**Affiliations:** 1 Department of Physiology, CIMUS, University of Santiago de Compostela-Instituto de Investigación Sanitaria (IDIS), Santiago de Compostela, Spain; 2 CIBER Fisiopatología de la Obesidad y Nutrición (CIBERobn), Instituto de Salud Carlos III, Santiago de Compostela, Spain; 3 Faculty of Biology, Medicine and Health, University of Manchester, AV Hill Building, Manchester, UK; 4 Instituto Maimónides de Investigación Biomédica de Córdoba (IMIBIC)/University of Córdoba/Reina Sofia University Hospital, Edificio IMIBIC, Avda. Menéndez Pidal s/n, 14004 Córdoba, Spain; 5 Department of Medical Sciences, Faculty of Medicine, Ciudad Real, Spain

## Abstract

During their lifetime, females are subjected to different nutritional and hormonal factors that could increase the risk of obesity and associated comorbidities. From early postnatal periods until the postmenopausal phase, exposure to over nutrition, high-energy diet and oestrogen deficiency, are considered as significant obesity risk factors in women. In this study, we assessed how key transitional life events and exposure to different nutrition influence energy homeostasis in a rat model. Specifically, we assessed the sequential exposure to postnatal over nutrition, high-fat diet (HFD) after weaning, followed later by ovariectomy (OVX; as a model of menopause). Each obesity risk factor increased significantly body weight (BW) and adiposity, with additive effects after sequential exposure. Increased energy intake in both HFD and/or OVX groups, and decreased locomotor activity and energy expenditure after OVX can explain these metabolic changes. Our study also documents decreased lipogenic pathway in mesenteric adipose tissue after HFD and/or OVX, independent of previous postnatal programming, yet only HFD evoked this effect in liver. In addition, we report an increase in the expression of the hepatic PEPCK depending on previous metabolic status. Overall, our results identify the impact of different risk factors, which will help in understanding the development of obesity in females.

Obesity and its related metabolic diseases are nowadays considered a worldwide epidemic affecting over 600 million adults^1^. Of note, the incidence of obesity is higher in women^2^. The increasing prevalence of obesity, and consequently a growing morbidity and mortality, results from a constant and complex relation between predisposing genes and environmental factors^3^,^4^. Although the risk of obesity and metabolic syndrome increases during adult life due to excessive consumption of energy-dense foods and sedentary lifestyles, the predisposition to the development of metabolic diseases begins during prenatal and postnatal periods. This stage is crucial for the establishment of the hypothalamic set point regulating energy homeostasis^5^,^6^,^7^.

From early life, mammals are subjected to constant metabolic adaptation. The early-life plasticity allows offspring the potential to flourish in their new environment. However, inappropriate adaptation during early life may predispose to obesity and metabolic diseases in later life^8^. Because hypothalamic development mostly takes place after birth in rodents, a classic model of postnatal nutritional “programming” is the manipulation of rat litter size in the first days of life, which mimics altered nutritional conditions during the last trimester of human gestation^9^. Rats growing up in small litters have more access to milk and, consequently, display hyperphagia and excess weight. Notably, this phenotype and its associated metabolic disturbances, such as hyperleptinemia and hyperinsulinemia, are maintained throughout their lives^10^,^11^,^12^,^13^,^14^. Moreover, postnatal overfeeding animals showed an enhanced response to orexigenic hormone ghrelin, which could partly explain the obese phenotype^15^.

Likewise, postnatal over nutrition condition animals to be more susceptible to metabolic diseases when later fed with high-energy diets^10^,^11^,^16^,^17^,^18^. In fact, postnatal programming accentuates obesity, insulin resistance and glucose intolerance induced by feeding with a high-fat diet (HFD)^16^. Also, long-lasting leptin resistance in the arcuate nucleus of the hypothalamus, as well as decreased brown adipose tissue (BAT) thermogenesis and fatty liver disease have been reported in other studies^10^. The impact of postnatal overfeeding and high fat diet as obesogenic risk factors goes beyond energy homeostasis, affecting other physiological functions. For example, it has been demonstrated recently that both factors have deleterious consequences on the female gonadotropin axis, especially when were combined with oestrogen deficiency^11^.

Oestrogen levels have an important role in regulating energy homeostasis. Experimental studies have shown conclusively that ovariectomy is associated with hyperphagia, reduced energy expenditure and increased adiposity and body weight, which can be reversed following oestrogen treatment^19^,^20^. Similar effects have been observed in humans, where impaired or deficient ovarian function leads to increased body weight and a higher risk (57%) of developing diabetes^21^. In line with this, women treated with low oestrogen doses in replacement therapy at menopause, exhibit a 35% lower incidence of diabetes in comparison with untreated women^22^. Oestrogen deficiency increases susceptibility to the deleterious effects of HFD while restoration of oestradiol at physiological concentrations prevents the metabolic changes associated with HFD consumption^23^,^24^,^25^,^26^. Among the potential mechanisms behind these effects elicited by oestrogen deficiency, inflammation and dyslipidemia are considered to play a major role^27^.

Overall, this evidence demonstrates the importance of each obesogenic risk factor. However, the relative contribution and/or potential for synergy are not well studied. The aim of the current paper is to determine the metabolic consequences of different obesogenic risk factors, postnatal overfeeding, HFD, and oestrogen deficiency, when applied alone or in combination at different developmental stages of female rats. Moreover, we explore changes in lipid and intermediary metabolism associated with these obesogenic factors.

## Results

### Sequential postnatal over feeding, high-fat diet and ovariectomy produces an obese phenotype

**Figure 1:**
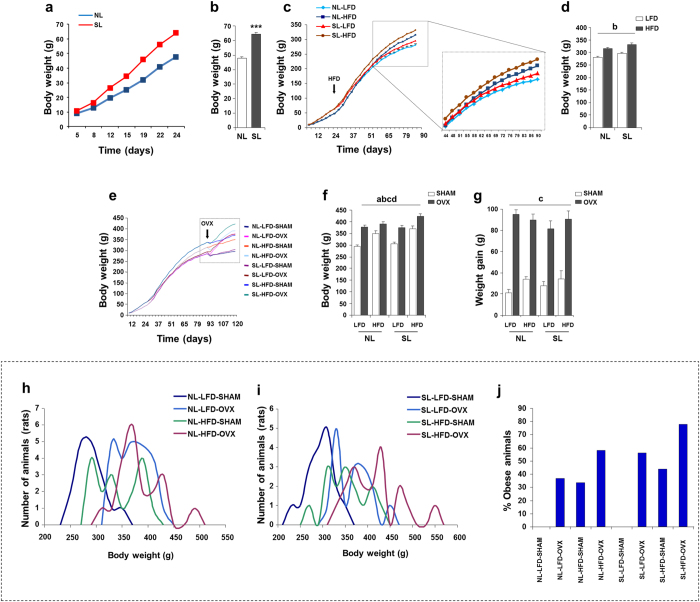
Postnatal over feeding, high-fat diet and ovariectomy all increase body weight. (**a**) Body weight from day 5 until weaning in NL (normal litter) and SL (small litter) animals. (**b**) Body weight at weaning (PND24) (n = 105–10/group). (**c**) Body weight until ovariectomy. (**d)** Body weight at PND90, before ovariectomy (n = 50–55/group). (**e**) Body weight during sequential exposure to all risk factors. (**f**) Body weight at PND120 (n = 16–20/group). (**g**) Body weight gain after ovariectomy. (**h**) Body weight distribution of NL animals at PND120. (**i**) Body weight distribution of SL animals at PND120. (**j**) Percentage of obese animals at PND120 (body weights ranging between the average ± 3 standard deviations of the control group (NL-LFD-SHAM)). Annotation indicates significant effect of a = postnatal over feeding, b = HFD, c = OVX, d = significant HFD-OVX interaction (ANOVA) and ***p < 0.001 (t-test). All data are expressed as mean ± SEM.

Our first goal was to study body weight accumulation from birth until weaning at postnatal day 24 (PND24). Animals were weighed from day 5, when a significantly higher weight was recorded in the small litter (SL) group because of postnatal over nutrition (Fig. 1a), and this difference increased through the lactation period (Fig. 1b). Once weaned, the animals were divided into two groups and fed diets with different fat content. Soon after, HFD-fed animals exhibited a higher body weight, and this effect was even greater in those animals that had previously been exposed to postnatal over nutrition (Fig. 1c and d). Thereafter, at postnatal day 90 (PND90) animals were ovariectomised in order to induce oestrogen deficiency. This sequential exposure allowed us to assess the individual influence of each obesogenic risk factor, and to uncover any interactions (Fig. 1e). So, although at postnatal day 120 (PND120), we found that each risk factor studied had a statistically significant effect on final body weight, the cumulative effect as unequal between groups. We found a statistically significant interaction between HFD and OVX. This reflected the fact that oestrogen deficiency did not have such a strong effect on the body weight of animals that had already been subjected to HFD (these animals showed a higher body weight already when they were ovariectomised; Fig. 1f). If observations were limited to the oestrogen-deficient period (Fig. 1g), ovariectomy elicited a significant body weight increase in all groups. The relevance of these changes in body weight was further assessed by assessing the number of animals that showed a marked increment in body weight; namely those with body weights ranging between the average ± 3 standard deviations of the control group (Normal Litter [NL]-Low fat diet [LFD]-Sham-operated [SHAM])^28^. Using this approach, we found a similar percentage of obese individuals following exposure to either HFD or OVX. Notably, sequential exposure to the three obesogenic stimuli led to marked body weight gain (up to 78% of animals are considered obese) (Fig. 1h–j).

### Sequential effects of obesity risk factors on body composition

**Figure 2:**
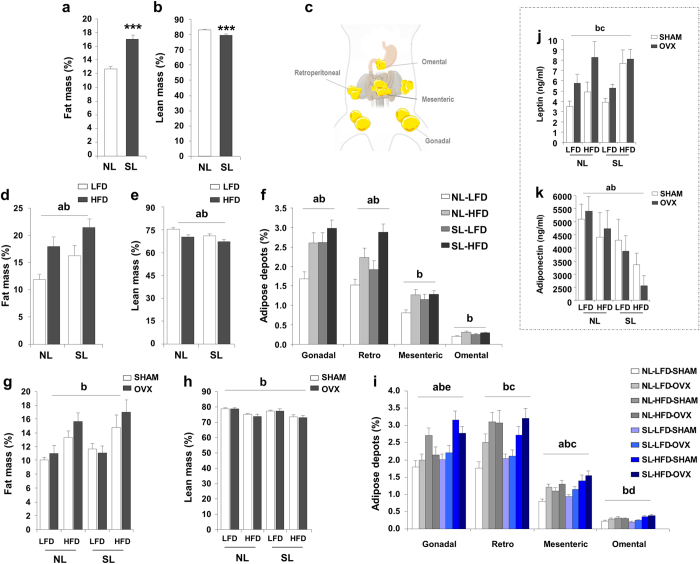
Significant changes in body composition. (**a**) Fat mass (%) at weaning (n = 25–32/group). (**b**) Lean mass (%) at weaning (n = 25–32/group). (**c**) Anatomy of major fat depots analised in this work. (**d**) Fat mass (%) at PND90 (n = 16–18/group). (**e**) Lean mass (%) at PND90 (n = 16–18/group). (**f**) Percentage contribution of different adipose depots at PND90 (n = 18–20/group). (**g**) Fat mass (%) at PND120 (n = 12–14/group). (**h**) Lean mass (%) at PND120 (n = 12–14/group). (**i**) Percentage contribution of different adipose depots at PND120 (n = 16–20/group). (**j**) Leptin serum levels (ng/ml). (**k**) Adiponectin serum levels (ng/ml). Annotation indicates significant effect of a = postnatal over feeding, b = HFD, c = OVX, d = significant postnatal over feeding-HFD interaction, e = significant HFD-OVX interaction (ANOVA) and ***p < 0.001 (t-test). All data are expressed as mean ± SEM.

The percentage of fat and lean mass was determined by nuclear magnetic resonance (NMR). We observed that, at PND24, animals over fed during lactation had a significant higher percentage of fat mass (Fig. 2a) and a lower percentage of lean mass (Fig. 2b). Also, HFD caused a very significant increment of fat mass (Fig. 2d) and a drop in lean mass (Fig. 2e) after weaning. The latter were more significant in animals previously subjected to postnatal programming. The HFD effect was evident in individual fat depots: gonadal, retroperitoneal, mesenteric and omental. Moreover, in gonadal and retroperitoneal depots, postnatal over feeding effect was observed too (Fig. 2f). At PND120, HFD increased total percentage of fat mass (Fig. 2g).and decreased lean mass (Fig. 2h), meanwhile, ovariectomy contributed to a greater increase in whole-body fat content, though this effect was mainly within, mesenteric adipose tissue (Fig. 2i).

These significant changes in body composition were correlated, as expected, with increased serum leptin and serum adiponectin levels. Thus, when animals were subjected to HFD and/or OVX circulating leptin levels increased significantly and additively (Fig. 2j), though the effect of neonatal over feeding on leptin was lower than expected when considering its effect on fat mass. In contrast, we observed a clear-cut and very potent decrease in adiponectin levels when animals were exposed to the obesogenic risk factors in a sequential fashion (Fig. 2k).

### High-fat diet and oestrogen deficiency promote feeding

**Figure 3:**
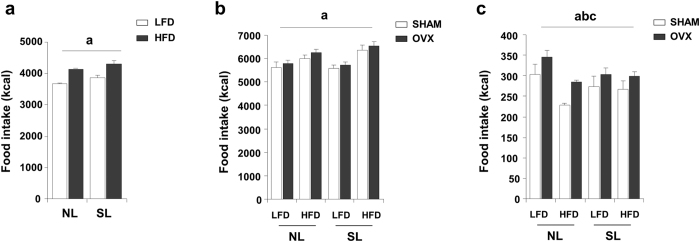
Increased energy intake under HFD and OVX conditions. (**a**) Cumulative food intake (kcal) at PND90 (n = 10 groups). (**b**) Cumulative food intake from weaning until sacrifice (n = 6 groups). (**c**) Cumulative food intake (kcal) during the last 15 days of the experiment, once a residual oestrogen effect had passed (n = 6 groups). Annotation indicates significant effect of a = HFD, b = OVX, c = significant postnatal over feeding-HFD interaction (ANOVA). All data are expressed as mean ± SEM.

Next, we assessed whether the changes in body weight and body composition could reflect changes in food intake. Animals subjected to HFD ingested significantly more energy (kcal) both at PND90 (Fig. 3a) and at PND120 (Fig. 3b). However, a significant effect of OVX on food intake was only observed if cumulative kcal was measured during last 15 days before sacrifice (between PND105 and PND120, when the effect of OVX in oestrogen levels was clear) (Fig. 3c).

### Oestrogen deficiency decreases locomotor activity and energy expenditure

**Figure 4:**
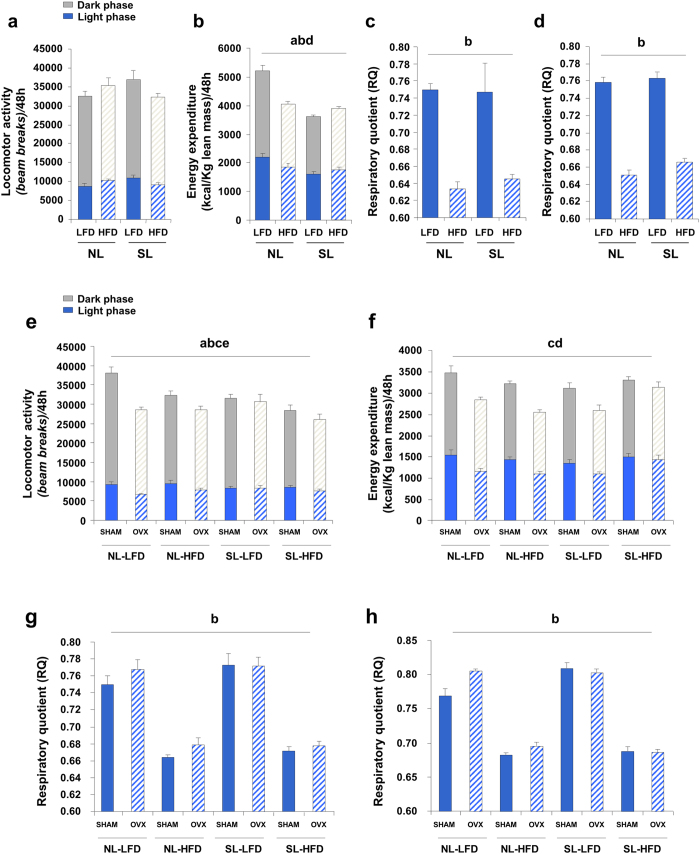
Energy expenditure changes in obesogenic conditions. (**a**) Locomotor activity (beam breaks/48 h) at PND90 (n = 9/group). (**b**) Energy expenditure (kcal/kg lean mass/48 h) at PND90 (n = 9/group). Respiratory quotient during the light phase **(c)** and dark phase **(d)** at PND90 (n = 9/group). (**e**) Locomotor activity (beam breaks/48 h) at PND120 (n = 9/group). (**f**) Energy expenditure (kcal/kg lean mass/48 h) at PND120 (n = 9/group). Respiratory quotient during the light phase **(g)** and dark phase **(h)** at day 90 (n = 9/group). Annotation indicates significant effect of a = postnatal over feeding, b = HFD, c = OVX, d = significant postnatal over feeding-HFD interaction, e = significant postnatal over feeding-OVX interaction (ANOVA). All data are expressed as mean ± SEM.

In order to investigate whether body weight and composition changes also are dependent on energy expenditure, rats underwent indirect calorimetry and activity analysis before ovariectomy. At PND90, no significant differences were found in locomotor activity (Fig. 4a). However, energy expenditure (kcal/kg lean mass), exhibited a strong interaction between the postnatal over feeding and HFD, with a significant decrease evident during the dark phase. HFD reduced energy expenditure in NL but not in SL animals, since these already showed a decrease in energy expenditure (Fig. 4b). For a deeper analysis of the data^29^,^30^, we applied more stringent statistical tests (general linear model [GLM] and analysis of covariance [ANCOVA]) (Supplemental Data Fig.1S). Thus, considering lean tissue weight as a covariate, results showed that the differences observed were dependent lean mass and not on the individual risk factors studied (although we found that postnatal over feeding and HFD modified the effect of lean tissue). A decrease in respiratory quotient (RQ) was observed in both light (Fig. 4c) and dark phase (Fig. 4d) when animals were fed with HFD and this was independent of postnatal programming. When we performed indirect calorimetry studies at PND120, we observed a statistically significant effect of postnatal over feeding and HFD, and extremely significant OVX effect on locomotor activity (Fig. 4e). Thus, as obesogenic factors were added, locomotor activity fell. Notably, in oestrogen-deficient conditions, locomotor activity was reduced irrespective of the other factors. In other words, only the sum of postnatal over feeding and HFD, could reduce locomotor activity level to the same extent as ovariectomy alone.

We observed that the OVX produced a highly significant decrease on energy expenditure in all groups, with the exception of SL-HFD rats. On the other hand, we saw that there was a statistically significant interaction between postnatal over feeding and HFD. So, the HFD effect on energy expenditure depends on previous neonatal programming. In NL-HFD animals, energy expenditure decreased. This was not observed in SL-HFD rats, and even the effect of OVX was much less marked. The differences were observed mainly in the light phase (Fig. 4f). We analysed by ANCOVA the effect of lean mass on energy expenditure, considering lean mass as covariate, and observed that the OVX effect was again dependent on the amount of lean tissue.

Finally, we determined RQ at PND120. We observed that only HFD had a significant effect on RQ, during both the light (Fig. 4g) and dark phase (Fig. 4h). However, when we analysed the light and dark-phase data together, we obtained a statistically significant effect due to OVX only in NL animals (significant interaction).

### Tissue specific effects on lipid metabolism: mesenteric adipose tissue versus liver

Proteomic results and their subsequent confirmation by Western blot (Fig. S2, Table 1a-1b and 2, Supplemental data), led us to focus our study on the expression of proteins involved in intermediary metabolism (Fig. S3, Supplemental data).

**Figure 5:**
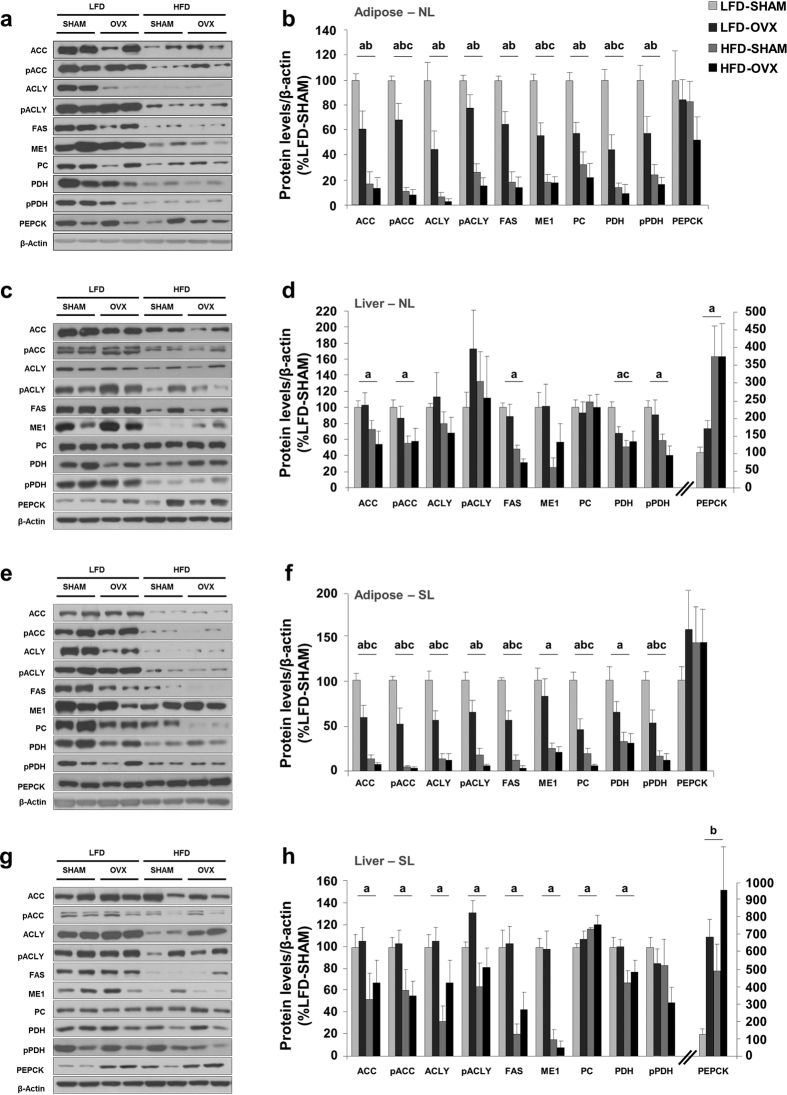
High-fat diet and ovariectomy decreased mesenteric adipose tissue *de novo* lipogenic pathway in normally fed (NL) and over fed (SL) postnatal animals, while obesogenic conditions increased PEPCK expression. (**a**) Representative images of Western blots in NL mesenteric adipose tissue. (**b**) NL mesenteric adipose tissue protein levels (n = 6–8/group). (**c**) Representative images of Western blots in NL liver. (**d**) NL liver protein levels (n = 6–8/group). (**e**) Representative images of Western blots in SL mesenteric adipose tissue. (**f**) SL mesenteric adipose tissue protein levels (n = 6–8/group). (**g**) Representative images of Western blots in SL liver. (**h**) SL liver protein levels (n = 6–8/group). Cropped gel images from not adjacent samples are shown. All samples derive from the same experiment and all gels/blots were processed in parallel. Annotation indicates significant effect of a = HFD, b = OVX, c = significant HFD-OVX interaction (two-way ANOVA). All data are expressed as mean ± SEM.

The expression of proteins involved in metabolic homeostasis was measured in tissues from animals at PND120. To simplify understanding, NL and SL analyses are shown separately. In mesenteric adipose tissue of NL animals, we observed a significant reduction in expression of each of the enzymes we analysed following exposure to both HFD and OVX (the exception was phosphoenolpyruvate carboxykinase, PEPCK, which did not change). Because of the marked effect exerted by exposure to HFD alone, no further effect of concomitant exposure to OVX was measured (Fig. 5a and b). The specificity of these results was tested in liver extracts from the same animals. Here, less effect of HFD was observed, with acetyl-CoA carboxylase (ACC), pACC, fatty acid synthase (FAS), pyruvate dehydrogenase (PDH) and pPDH all decreased, while PEPCK was increased (Fig. 5c and d). On the other hand, as in NL animals, similar pattern expression was observed in mesenteric adipose tissue of postnatal over fed animals (SL). Thus, both HFD and OVX factors caused greater decrease in expression in SL than in NL animals, suggesting postnatal over feeding enhances the inhibitory effect of the other obesity risk factors. No expression changes were observed in PEPCK in adipose tissue of SL animals (Fig. 5e and f). The enhanced effect of postnatal over feeding was also observed in the liver of animals subjected to HFD, independent of ovariectomy. Moreover, in the liver of SL animals an increase PEPCK expression was observed in oestrogen deficiency (Fig. 5g and h).

## Discusion

Over the last years, the incidence of obesity has reached epidemic proportions worldwide, with notable increases in children and postmenopausal women^31^. Although the involvement of genetic and environmental factors is accepted, there is a consensus that events taking place at early postnatal periods may play a particularly major role in the susceptibility to develop obesity in adulthood. In this context, large-scale animal studies addressing the impact of various forms of metabolic/nutritional stress^11^,^32^,^33^ have reported that over nutrition at early postnatal stages exerts potent effects on sensitivity to HFD, on adiposity and on metabolic homeostasis in adulthood. However, it should be noted that most of these studies were conducted in male animals, while much less data are available for females. To overcome this lack of knowledge, we performed our studies in female rats. In addition, our setting was designed to address a critical issue in the development of obesity, namely the impact of deficient ovarian function. Our data suggest that the abnormalities observed in ovariectomised animals are significantly increased when animals are previously exposed to early postnatal over nutrition and/or exposed to HFD in adulthood.

Previous studies have shown that litter size provides an accurate model for postnatal programming by early over nutrition. Postnatal over-fed animals are hyperphagic and this behaviour remains in adulthood^10^,^34^. In keeping with previous reports, our results showed a greater weight and fat-mass gain when animals were exposed to a series of obesity risk factors. Although this might have been predicted, our data provide direct evidence that this is the case. Thus, the marked increase in adiposity and weight gain in animals exposed to the three sequential factors indicates the relevance and detrimental effects of each and every together in energy homeostasis.

It is well established that ovarian oestrogens have a major role in metabolic homeostasis. States of ovarian deficiency rapidly lead to insulin resistance and increased adiposity and body weight. In addition, there is an accepted physiological role for oestrogens, since caloric intake varies across the ovarian cycle in women and female rodents^19^,^20^,^31^. Thus, surgical removal of the ovaries in rats produces marked hyperphagia; effect that we also could observe in our model once ovarian steroids were depleted. Moreover, by acting centrally, oestrogens markedly increase energy expenditure as well as regulating lipid and metabolic homeostasis^20^,^35^. Despite this wealth of knowledge, some relevant questions remain unclear. Even though both over nutrition and HFD are associated to increased body weight in animals of both sexes, there are marked gender-dependent differences regarding effects on adiposity and body-fat distribution. Although some of these differences are set up at early in development, our data showed that sequential ovariectomy enhanced the over feeding and HFD-effect on adiposity, indicating that oestrogen deficiency leads to increased risk to early exposure of obesogenic factors. It should be noted that the amount of visceral fat is a major risk for metabolic syndrome and insulin resistance. We also found dramatic changes in leptin and adiponectin levels. Notably, animals exposed to the three risk factors showed very low levels of adiponectin, which is considered as one of the best biomarkers of cardiovascular risk in obesity. Furthermore, low oestrogen levels lead to an accentuated prevalence of metabolic diseases in women^31^,^36^,^37^.

We further assessed the changes in energy balance related to the different obesogenic conditions. We noted hyperphagia after ovarian removal, while some studies have concluded differences in body weight and composition in OVX animals can be explained due to decreased locomotor activity and energy expenditure, not by increased food intake^38^,^39^. Data produced recently have shown an action of oestradiol in the ventromedial nucleus of the hypothalamus (VMH), through oestrogen receptor alpha (ERα), which caused increased energy expenditure, elevated BAT thermogenesis and decreased respiratory quotient, leading to lower body weight^20^,^40^. Interestingly, this seems to be a gender- and nutrient-dependent effect since, when fed a normal diet, female (but not male) VMH ERα/SF1-null (steroidogenic factor 1, SF1) mice showed a moderate increase in body weight and visceral adiposity, which was aggravated when they were fed a HFD^35^. Taken together, these data imply a major role of the VMH in the control of energy balance in response to changes in oestrogen levels. The relevance of these neurons was further reinforced by recent data unmasking a particular subset of neurons that are oestrogen-responsive and influence female locomotion independently of other parameters involved in energy expenditure such as BAT thermogenesis^41^,^42^.

Our results show that at an early stage of obesity (PND90) neither the postnatal overfeeding, nor HFD alone had significant effects on the locomotor activity. In contrast oestrogen deficiency alone had a great impact by decreasing locomotor activity. In fact, only the additive effect of postnatal over feeding and HFD produced a comparable result, though the effect was maximal when the animals were exposed to the three obesity risk factors. Whether postnatal over nutrition, HFD and oestrogens act on the same subset of VMH neurones described by Correa *et al*.^41^ or not, remains to be established. Interestingly, OVX animals showed a significant reduction in energy expenditure apart from SL-HFD-OVX group. At first glance, this may appear counterintuitive since these animals presented the highest BW and adiposity. We speculate that these animals may have reached a situation where the organism is maintaining certain degree level of energy expenditure in an attempt to compensate a major pathological damage^43^. On the other hand, HFD-fed animals metabolize primarily fats, to the detriment of carbohydrates (greater RQ), that in the long run could be related to a state of peripheral insulin resistance, and a decrease in glucose oxidation^44^. In addition, plasma lipid concentration modifies metabolic flexibility, by impairing insulin-stimulated glucose disposal rate and by enhancing adipose tissue lipid storage capacity^45^. Meanwhile, oestrogen deficiency increased RQ in NL animals. A reduced fat oxidative capacity after OVX may contribute to a greater adiposity too, mainly in intra-abdominal depots. Although a significant effect was not observed in SL animals, probably because these animals showed a slightly higher RQ value by themselves, we cannot rule out that small variations in fat oxidative capacity over time might influence a positive energy balance. Taken together, our data further confirm a major effect of oestrogen deficiency on energy homeostasis and uncovered the relevance of previous exposure to other obesity risk factors. Indeed, in only 15 days, once residual oestradiol levels were eliminated completely (PND105 to PND120); ovariectomy caused a great impact on key metabolic parameters, such as body weight, energy intake, locomotor activity and energy expenditure. This worsened if animals had been previously subjected to postnatal over nutrition and/or HFD.

Finally, and considering our data showing marked changes in adiposity and RQ, we analysed intermediary metabolic pathways in liver and adipose tissue at PND120. Our data showed a significant reduction of *de novo* lipogenesis program after obesogenic conditions. This adaptation would reduce a costly metabolic pathway, in a situation of abundant adiposity. An increase in the availability of lipids promotes excessive oxidation by causing the uncoupling between β-oxidation and the Krebs cycle, resulting in the generation of a large amount of partially oxidized lipids; which may interfere with insulin signalling and glucose transport^45^exacerbating the insulin resistance condition. In the same context, both HFD and OVX animals exhibit higher expression of PEPCK levels in the liver. The influence of HFD and oestrogens in the regulation of glucose homeostasis has been studied extensively^46^,^47^,^48^. In the present work, we showed a significant increase in PEPCK expression, after HFD or oestrogen deficiency conditions. An increase of PEPCK in the liver suggests activation of gluconeogenesis. So, despite greater available energy in a pre-diabetes status, gluconeogenesis might be increased in order to guarantee nutrients to the cells. On the other hand, PEPCK has a key role in glyceroneogenesis^49^. So, activated PEPCK will increase the incorporation of free fatty acids into triglycerides, which will be stored in adipose tissue, aggravating the obese phenotype^50^. Further studies aimed at gaining mechanistic insight are warranted.

In summary, our data show that oestrogen deficiency impairs metabolic homeostasis and enhances the deleterious effect of a previous obesogenic environment. Besides, our study is the first to show the aggravated impact of the sequential action of obesity risk factors on intermediary metabolism. These findings suggest that this model may help to understand the metabolic complications that accompany the development of oestrogen deficiency whether of physiological or pathological onset.

## Material And Methods

### Animals

Pregnant female Sprague–Dawley rats (300–350 g) (Animalario Central, USC, Spain) were housed at 23 °C ± 2 °C under a 12 h light, 12 h dark cycle. Animals were allowed free access to standard chow and tap water. On day one of life, neonates were distributed randomly among the mothers. The litter size was adjusted to induce early postnatal over- or normal feeding; being either, small litters (SL) with 3–4 pups in each litter (over feeding), or normal litters (NL, control) with 12 pups per litter (normal feeding), as described previously^11^,^15^. Animals were separated from their mothers at postnatal day (PND) 24 in order to ensure complete weaning and divided in two groups. One group was fed with high-fat diet (HFD; D12451, 45 kcal percent fat, 35 kcal percent carbohydrate, and 20 kcal percent protein; 4.73 kcal/g) and the other with low-fat diet (LFD, control; D12450B, 10 kcal percent fat, 70 kcal percent carbohydrate, and 20 kcal percent protein; 3.85 kcal/g; Research Diets, Inc., New Brunswick, NJ). At PND90, animals were subdivided again, with one group subjected to bilateral ovariectomy, as a model of cessation of ovarian secretions to mimic menopause, under ketamine–xylazine anaesthesia. Sham-operated animals served as corresponding controls. Uterine atrophy was used to assess *in vivo* oestrogen status. At PND120, all animals were euthanized by decapitation. Trunk blood was collected and centrifuged, and serum was stored at −20 °C. Tissues of interest were snap-frozen in liquid nitrogen immediately after resection and stored at −80 °C until their use. In the study presented here, we used only female rats. Animals were group housed (4 animals per cage), were weighed twice per week and food intake was measured between days 27 and 120. An overview of the experimental design is provided in Supplemental Figure 4S. The Ethics Committee of the University of Santiago de Compostela approved the protocols, and experiments were performed in agreement with the rules of laboratory animal care and international law on animal experimentation.

### Body composition

Determination of body composition was done at PND24, PND90 and PND120 by nuclear magnetic resonance (NMR) (Echo Medical Systems, Houston, TX). Body composition was measured two times in each rat, and results represent means of both measurements, expressed as a percentage of total body weight. Moreover, at PND90 and PND120 rats were dissected, and the weights of different fat depots (gonadal, retroperitoneal, mesenteric and omental) were determined.

### Energy balance

Animals were monitored in a custom, 12-cage indirect calorimetry and locomotor activity monitoring system (LabMaster, TSE Systems, Germany). Rats were acclimated for 48 hr to the test chambers and then were monitored for an additional 48 hr before ovariectomy (PND90) and euthanasia (PND120). Measurements were taken every 30 minutes.

### Serum measurements

Serum leptin and adiponectin concentrations were analysed by specific double-antibody RIA for rat leptin (Linco Research, Cat. #RL-83K) and mouse adiponectin (Linco Research, Cat. #MADP-60HK). All samples were assayed in duplicate within one assay and results were expressed in relation to leptin or adiponectin standards.

### Western blotting

Liver and mesenteric adipose tissue were homogenized and subjected to SDS-PAGE on 6.5% polyacrylamide gels and electrotransferred on a polyvinylidene fluoride membrane (Millipore). Membranes were probed with the following antibodies: ACC, pACC (Ser79) (Millipore), ATP citrate lyase (ACLY), pACLY (Ser454), PDH (Cell signalling), FAS (H-300), pyruvate carboxylase (PC) (Santa Cruz), malic enzyme 1 (ME1), pPDH (Ser293), PEPCK (Abcam) and β-actin (Sigma). Detection of proteins was performed using HRP-conjugated secondary antibodies (Dako Denmark) and an enhanced chemiluminescence reagent (Thermo scientific). Optical densities of the immunoreactive bands were measured using ImageJ 1.40 g analysis software. Values were expressed relative to β-actin levels. We used 6 to 8 animals per experimental group.

### Statistical analysis and data presentation

The experiments involving two groups were analysed by two-tailed unpaired Student’s t-test. In the experiments involving four or eight groups, the data were analysed by two-way or three-way ANOVA (analysis of variance), respectively. ANCOVA was used to analyse energy expenditure data. Previously, normal distribution (Shapiro-Wilk test) and homoscedasticity (Barlett’s test) were tested. Data are expressed as mean ± SEM and analysed using Graph Pad Prism 5 for Windows (San Diego, California, USA) and the R-project 2.13.1. A value of *P* < 0.05 was considered as being significant.

## Additional Information

**How to cite this article**: Novelle, M. G. *et al*. Sequential Exposure to Obesogenic Factors in Females Rats: From Physiological Changes to Lipid Metabolism in Liver and Mesenteric Adipose Tissue. *Sci. Rep.* **7**, 46194; doi: 10.1038/srep46194 (2017).

**Publisher's note:** Springer Nature remains neutral with regard to jurisdictional claims in published maps and institutional affiliations.

## Supplementary Material

Supplemental Data
