# Supplementary material for: Sequential Exposure to Obesogenic Factors in Females Rats: From Physiological Changes to Lipid Metabolism in Liver and Mesenteric Adipose Tissue
Source: Sci Rep. 2017 Apr 7;7:46194. doi: 10.1038/srep46194 (PMC5384043; doi:10.1038/srep46194)
Supplement: Supplemental Data [file srep46194-s1.pdf]

ONLINE DATA SUPPLEMENT

Sequential Exposure to Obesogenic Factors in Females Rats: From Physiological Changes to Lipid Metabolism in Liver and Mesenteric Adipose Tissue

Marta G. Novelle[1,4,5, *], María J. Vázquez[2,4], Juan R. Peinado[3], Kátia D. Martinello[1,4], Miguel López[1,4], Simon M. Luckman[5], Manuel Tena-Sempere[2,4], María M. Malagón[2,4], Rubén Nogueiras[1,4] and Carlos Diéguez[1,4, *].

[1]Department of Physiology, CIMUS, University of Santiago de Compostela-Instituto de Investigación Sanitaria (IDIS), Santiago de Compostela, Spain,

[2]Instituto Maimónides de Investigación Biomédica de Córdoba (IMIBIC)/University of Córdoba/Reina Sofia University Hospital, Edificio IMIBIC, Avda. Menéndez Pidal s/n, 14004 Córdoba, Spain

[3]Department of Medical Sciences, Faculty of Medicine, Ciudad Real, Spain.

[4]CIBER Fisiopatología de la Obesidad y Nutrición (CIBERobn), Instituto de Salud Carlos III, Santiago de Compostela, Spain,

[5]Faculty of Biology, Medicine and Health, University of Manchester, AV Hill Building, Manchester, UK

**** corresponding-author:***

Marta G. Novelle, PhD and

Carlos Diéguez, PhD, MD

Department of Physiology, CIMUS

University of Santiago de Compostela-Instituto de Investigación Sanitaria (IDIS)

Avd. Barcelona s/n, Santiago de Compostela, Spain

Phone: +34647344282

e-mail:marta.novelle@manchester.ac.uk or marta.garrido@usc.es and carlos.dieguez@usc.es

Supplementary Figures and Tables

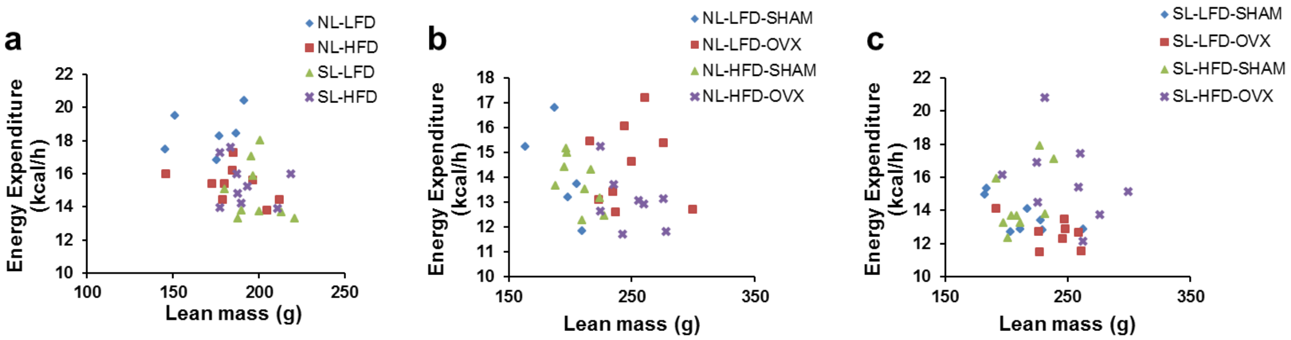

Fig 1S. a. Energy expenditure (kcal/h) in relation to lean body mass at PND90. **b**. Energy expenditure (kcal/h) in relation to lean body mass at PND120 in NL animals. **c**. Energy expenditure (kcal/h) in relation to lean body mass at PND120 in SL animals.

Molecular differences in mesenteric adipose tissue. Proteomic studies.

Approximately 800 spots were observed in the gels prepared from mesenteric adipose tissue extracts of animals subjected to postnatal over feeding and HFD (killed at 90 day). Comparative proteomic analysis of NL-LF animals *versus* SL-HFD animals showed 14 different proteins. The MALDI-TOF results are displayed in table 1a and table 1b. Each protein was identified by a spot number and indicated on 2D gels (Fig. 2Sa). One of these identified proteins was ATP-citrate lyase (ACLY) (Fig. 2Sb). To validate the results obtained we analysed the ACLY expression by Western blotting and confirmed that HFD decreased its expression significantly, regardless of previous postnatal over feeding (Fig. 2Sc). When we compared proteome of NL-HFD *versus* SL-LFD animals, 7 different spots were found and identified by mass spectrometry (Fig. 2Sd). The result of this identification is detailed in table 2. To validate the data we selected pyruvate dehydrogenase (PDH) and pyruvate carboxylase (PC) as proteins to analyse by Western blotting. In proteomic studies, both enzymes were decreased in NL-HFD animals (Fig. 2Se) and Western blot expression confirmed this result. Thus, animals subjected to HFD showed a statistically significant decrease in PDH (Fig. 2Sf) and PC expression (Fig. 2Sg). No differences were observed due to postnatal over feeding.

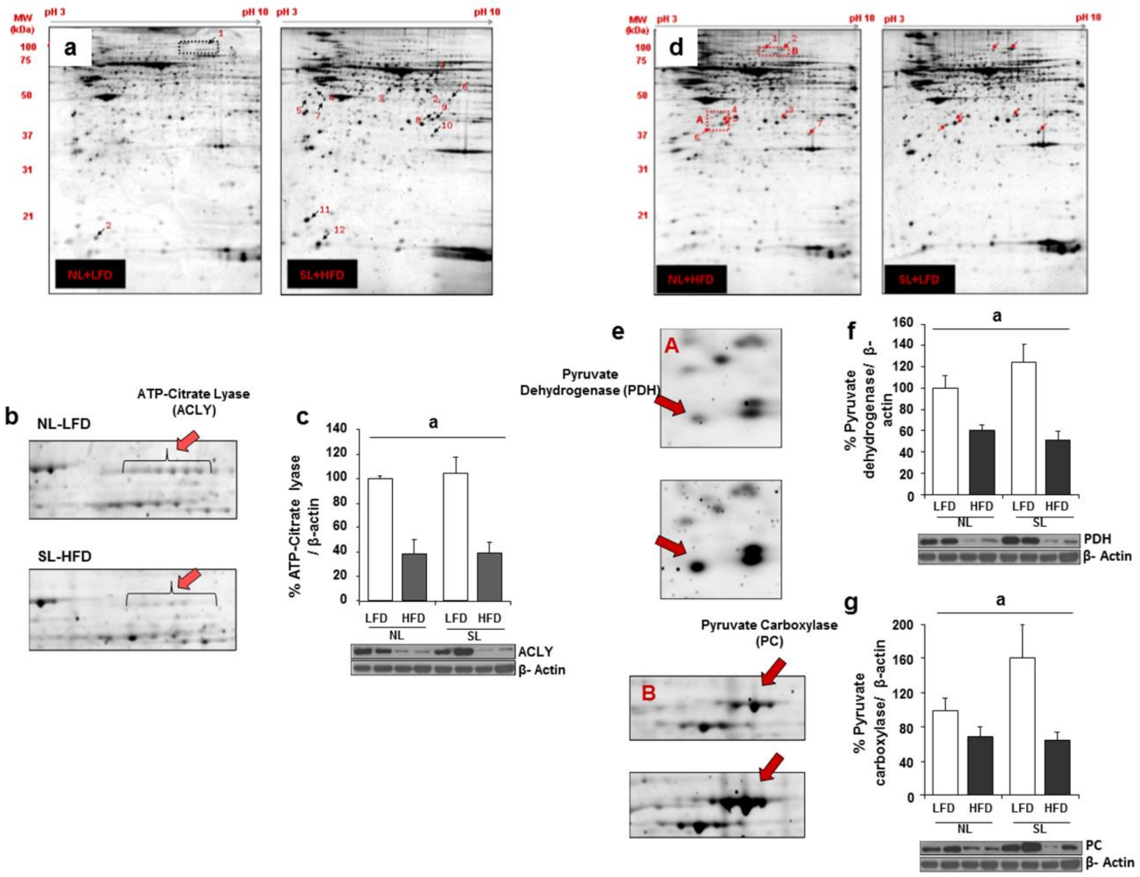

Figure 2S. Search for proteins involved in the pathophysiology of obesity. a. 2-D PAGE of mesenteric adipose tissue from NL-LFD and SL-HFD animals sacrificed at PND90. Differentially expressed proteins between the two groups are indicated with arrows. The numbers correspond to the spot numbers are listed in Table 1a (NL-LFD) and 1b (SL-HFD). **b.** Magnification of the boxed region of the 2-D PAGE gels, showing ACLY. **c.** ACLY levels in mesenteric adipose tissue (n=6-8/group) and representative images of Western blots for ACLY. **d.** 2-D PAGE of mesenteric adipose tissue from NL-HFD and SL-LFD animals sacrificed at PND90. Differentially expressed proteins between the two groups are indicated with arrows. The numbers correspond to the spot numbers listed in Table 2. **e.** Magnification of the boxed region of the 2-D PAGE gels, showing PDH (A in red) and PC (B in red). **f.** PDH levels in mesenteric adipose tissue and representative images of Western blots (n=6-8/group). **g.** PC levels in mesenteric adipose tissue and representative images of Western blots (n=6-8/group). Annotation indicates significant effect of a=HFD (two-way ANOVA). All data are expressed as mean ± SEM.

Table 1a. List of proteins identified by MALDI-TOF in mesenteric adipose tissue. Down- regulated proteins in SL-HFD vs NL-LFD animals. Spot number is indicated on the NL-LFD gel.

Spot	Protein	Accesion number	MW(kDa)/pI	Pep. d	Fold change	P
1	*ATP citrate lyase iso. 2*	NP_001104565	112.1/7.1	16	1.83	0.02
2	*Coactosin like1*	NP_001101922	16.1/5.3	10	2.26	0.04

Table 1b. List of proteins identified by MALDI-TOF in mesenteric adipose tissue. Up-regulated proteins in SL-HFD vs NL-LFD animals. Spot number is indicated on the SL-HFD gel.

Spot	Protein	Accesion number	MW (kDa)/pI	Pep. d	Fold change	P
1	*Cholesterol esterasase*	AAA88507	62.4/6.3	16	**No sign.**	
2	*Selenium binding protein 1*	NP_543168	53.1/6.1	31	1.40	0.01
3	*Cytokeratin 8*	EDL787221	52.7/5.5	29	1.90	0.02
4	*Vimentin. isoform CRA_b*	NP_112402	44.6/4.7	30	2.46	0.04
5	*Vimentin*	NP_112402	44.6/.4.7	28	3.08	0.001
6	*Isocitrate dehydrogenase 1*	NP_113698	47.1/6.5	22	2.21	0.001
7	*Apolipoprotein A IV*	P_036869	44.5/5.2	28	2.39	0.02
8	*Lipocortin 1*	NP_037036	39.1/7.0	25	2.03	0.03
9	*Aldo-keto reductase. family 1B1*	AAH62034	36.2/6.3	21	2.60	0.04
10	*Nucleoside phosphorylase*	NP_001099501	24.5/6.6	13	1.99	0.05
11	*Synuclein gamma*	EDL88878	13.1/4.7	14	2.44	0.05
12	*Lectin*	NP_063969	15.2/5.1	11	2.26	0.05

Table 2. List of proteins identified by MALDI-TOF in mesenteric adipose tissue. Down-regulated proteins in NL-HFD vs SL-LFD animals. Spot number is indicated on the NL-HFD gel.

Spot	Protein	Accesion number	MW (kDa)/pI	Pep. d	Fold change	P
1	*Albumin precursor*	GI 158138568	70.7/6.1	21	1.57	0.05
2	*Pyruvate Carboxilase*	NP_036876.2	130.4/6.3	47	1.61	0.03
3	*Ester hydrolase C11*	NP_001014228.1	35.4/6.1	12	1.63	0.04
4	*Gprotein beta1 subunit*	AAD00650.1	37.8/5.7	9	1.61	0.04
5	*Guanine nucleotide binding protein*	AAZ23824.1	35.7/5.9	11	1.60	0.01
6	*Pyruvate dehydrogenase*	NP_001007621	39.3/6.2	13	1.68	0.05
7	*Carbonyl reductase*	AAI05894.1	30.8/8.2	19	2.33	0.002

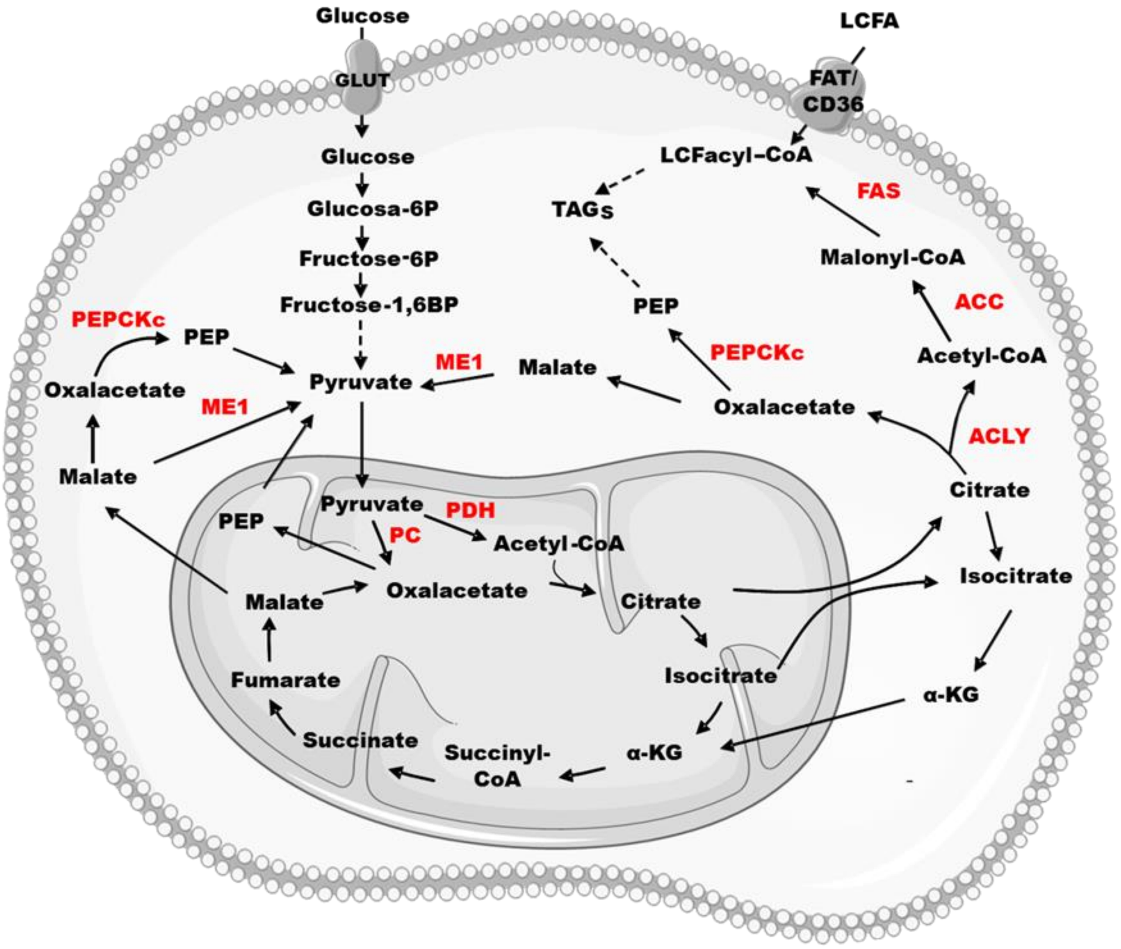

Figure 3S. Schematic representation of relevant metabolic intermediary pathways. **The enzymes chosen for Western blot expression studies are shown in red.** ACC, Acetyl CoA Carboxylase; pACC, phospho Acetyl CoA Carboxylase; ACLY, ATP-Citrate Lyase; pACLY, phospho-ATP-Citrate Lyase; FAS, Fatty Acid Synthase; ME1, Malic Enzyme 1; PC, pyruvate carboxylase; PDH, pyruvate dehydrogenase; pPDH, Phospho Pyruvate Dehydrogenase; PEPCKc, Phosphoenolpyruvate Carboxykinase Citoplasmatic.

Supplementary Material and Methods

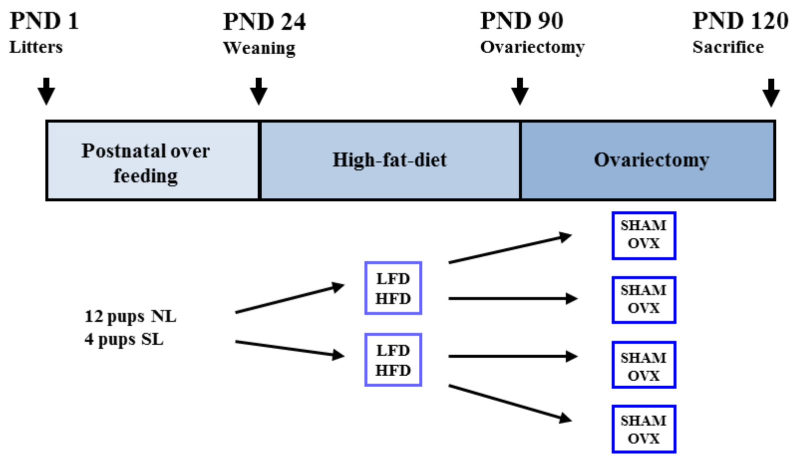

Figure 4S. Schematic representation of the different experimental groups included in this study. Interventions during lactation (NL/SL) or after weaning (nutritional LFD/HFD; hormonal SHAM/OVX) are shown in chronological order.

Proteomic studies

<u>Protein extraction</u>

For proteomic studies, mesenteric adipose tissue biopsies from a total of 12 animals were washed in PBS immediately after dissection and directly frozen in liquid nitrogen before storage at -80ºC. Samples were processed following a specific protocol previously optimized for adipose tissue [1,2].

<u>2-DE</u>

350 µg (50 µl) of protein from each animal, were diluted in 300 µl of Rehydratation Buffer and 0.8% of 3-10NL IPG buffer (GE Healthcare, Barcelona, Spain). Immobilized pH gradient strips (18 cm, pH 3–10 NL) were rehydrated overnight in a Ettan IPGPhor 3 System (GE Healthcare) [1,2]. Strips were equilibrated in SDS Equilibration Buffer containing 2% DTT for 15 min, followed by a 15-min wash with equilibration buffer containing 2.5% iodoacetamide. Thereafter, proteins were separated on 11% Tris-glycine gels using an Ettan Dalt Six device (GE Healthcare). After migration, gels were stained with Flamingo™ Fluorescent Gel Stain (Bio-Rad) and/or 0.1% Coomassie brilliant blue G-250, 10% ammonium sulfate, 2% phosphoric acid and 20% methanol. Both stains gave similar results.

MALDI-TOF-MS analysis

Spots were excised automatically using a ProPic station (Genomic Solutions, Huntingdon, UK) and subjected to MS analysis. MALDI-ToF-MS analyses were carried out according to a previously published protocol. [1,2].

Peak lists were submitted to the Mascot database in order to identify the proteins (Database, NCBInr 12012010 [10320603 sequences; 3520860234 residues]; taxonomy, Mammalia [757310 sequences]). Analysis was limited to peptides of six or more amino acids and maximum one missed cleavage site. Mass tolerance for precursor ions was set to 100 ppm and mass tolerance for fragment ions to 0.2 Da; oxidation of methionine was searched as variable modification and carbamidomethylation of cysteine was set as fixed modification. MS/MS data were also searched against the ENSEMBL *Rattus norvegicus* database using the open source software X!Tandem (http://www.thegpm.org) with similar settings to those employed for Mascot. Peptide false discovery rates (FDR) were determined by a target decoy approach using a reversed database concatenated to the parent forward database. A cutoff expectation value of ≤1.0 [significance threshold; expressed as the negative logarithm of E-value] was chosen for individual MS/MS spectra that resulted in a FDR of ≤1%.

Statistical analysis

2-DE gel analysis was performed by PDQuest software (Bio-Rad), version 8.0. Spot volume values were normalized in each gel by dividing the raw quantity of each spot by the total volume of all the spots included in the same gel. Data were log transformed to meet the requirements of a normal distribution and analyzed using the statistics tools included in the PDQuest software. Spots were verified visually to exclude artifacts and two-tailed unpaired Student's t-test was carried out on each spot after reevaluation of density with ImageJ 1.40g software.

Supplemental References

1 Peinado, J. R. *et al.* The stromal-vascular fraction of adipose tissue contributes to major differences between subcutaneous and visceral fat depots. *Proteomics* **10**, 3356-3366, doi:10.1002/pmic.201000350 (2010).

2 Peinado, J. R. *et al.* Proteomic profiling of adipose tissue from Zmpste24-/- mice, a model of lipodystrophy and premature aging, reveals major changes in mitochondrial function and vimentin processing. *Molecular & cellular proteomics : MCP* **10**, M111 008094, doi:10.1074/mcp.M111.008094 (2011).
